# Ankylosing spondylitis: acute/subacute vs. chronic iridocyclitis - a bidirectional two-sample Mendelian randomization study

**DOI:** 10.3389/fimmu.2023.1295118

**Published:** 2024-01-11

**Authors:** Hui Li, Yingying Xu, Qin Guo, Tiantian Zhang, Shufen Zhou, Meimei Wu, Yuanxiong Cheng, Chengshan Guo

**Affiliations:** ^1^ The Third Affiliated Hospital, The Third School of Clinical Medicine, Southern Medical University, Guangzhou, China; ^2^ Department of Rheumatology and Immunology, The People’s Hospital of Baoan Shenzhen, The Second Affiliated Hospital of Shenzhen University, Shenzhen, China; ^3^ Department of Intensive Care Medicine, Qingyuan People’s Hospital, Qingyuan, China; ^4^ Department of Respiratory and Critical Care Medicine, The Third Affiliated Hospital, Southern Medical University, Guangzhou, China

**Keywords:** Mendelian randomization, ankylosing spondylitis, iridocyclitis, causal relationship, single-nucleotide polymorphisms

## Abstract

**Background:**

Observational studies found associations between ankylosing spondylitis (AS) and iridocyclitis (IC), but the causality remained unconfirmed.

**Methods:**

We employed two-sample Mendelian randomization (MR) to investigate the bidirectional causal relationships between AS and IC. Single-nucleotide polymorphisms (SNPs) were chosen from the FinnGen database’s genome-wide association studies (GWAS) following a rigorous evaluation of the studies’ quality. Sensitivity analysis was performed to assess the potential influence of pleiotropy and heterogeneity on the MR findings.

**Results:**

Elevated genetic risk for AS showed positive causal effects on IC and its subtypes (IC, OR = 1.094, 95% CI = 1.035-1.157, *P* = 0.00156; Acute/Subacute IC, OR = 1.327, 95% CI = 1.266-1.392, *P* = 8.73×10^-32^; Chronic IC, OR = 1.454, 95% CI = 1.308-1.618, *P* = 5.19×10^-12^). Significant causal association was specifically observed between Acute/Subacute IC and AS (OR = 1.944, 95% CI = 1.316-2.873, *P* = 8.38×10^-4^). Sensitivity analysis suggested that horizontal pleiotropy was unlikely to influence the causality, and the leave-one-out analysis confirmed that a single SNP did not drive the observed associations.

**Conclusion:**

Our findings provide new proof of a positive causal relationship between AS and IC in the European population. Notably, it is Acute/Subacute IC, rather than IC as a whole or Chronic IC, that is associated with an elevated risk of AS. These results emphasize the significance of considering AS characteristics in the diagnosis of Acute/Subacute IC.

## 1 Introduction

Ankylosing spondylitis (AS) is a prevalent immune-related, chronic inflammatory condition primarily affecting the spine and sacroiliac joints (1, 2). It’s estimated to impact 9 to 30 individuals per 10000 worldwide (3). A multitude of studies across diverse populations and environments have associated AS with genetic predisposition (4, 5), autoimmunity and autoinflammation (6–8), gut microbiome (9, 10), and coexisting bone erosion and new bone formation (11, 12), contributing to a substantial global disease burden. AS often presents with extra-articular manifestations, including uveitis, psoriasis, and inflammatory bowel disease (IBD) (13, 14). Uveitis, occurring in up to 33% of AS cases, is the most common extra-articular manifestation (15).

Iridocyclitis (IC) stands as the most common form of uveitis (16). It often occurs in conjunction with other systemic medical disorders, including infections (17) and inflammatory diseases (18–20). Human leukocyte antigen (HLA)-B27-associated IC is the most commonly diagnosed form of IC and represents the largest entity of non-infectious uveitis worldwide (21). IC is frequently encountered in patients diagnosed with spondyloarthritis (22), and HLA-B27-positive Acute IC has a strong association with AS (RR = 6.80) (23). A cohort study found that 40% of individuals with IC, initially presenting to an ophthalmology emergency department without a prior diagnosis, had spondyloarthritis as the underlying cause (24). Investigating the relationship between AS and IC can enhance our understanding, potentially shedding light on early diagnosis for both. Nevertheless, the exact nature and direction of this association remain unexplained.

Mendelian randomization (MR) uses genetic variants as instrumental variables (IVs) to assess the causal effects between the exposure and the outcome, conquering the unobserved confounding and reverse causality inherent in observational studies (25). We conducted two-sample MR utilizing publicly available genome-wide association studies (GWAS) data to investigate the bidirectional causal effects between AS and IC, including IC subtypes.

## 2 Methods

### 2.1 Study design

**Figure 1:**
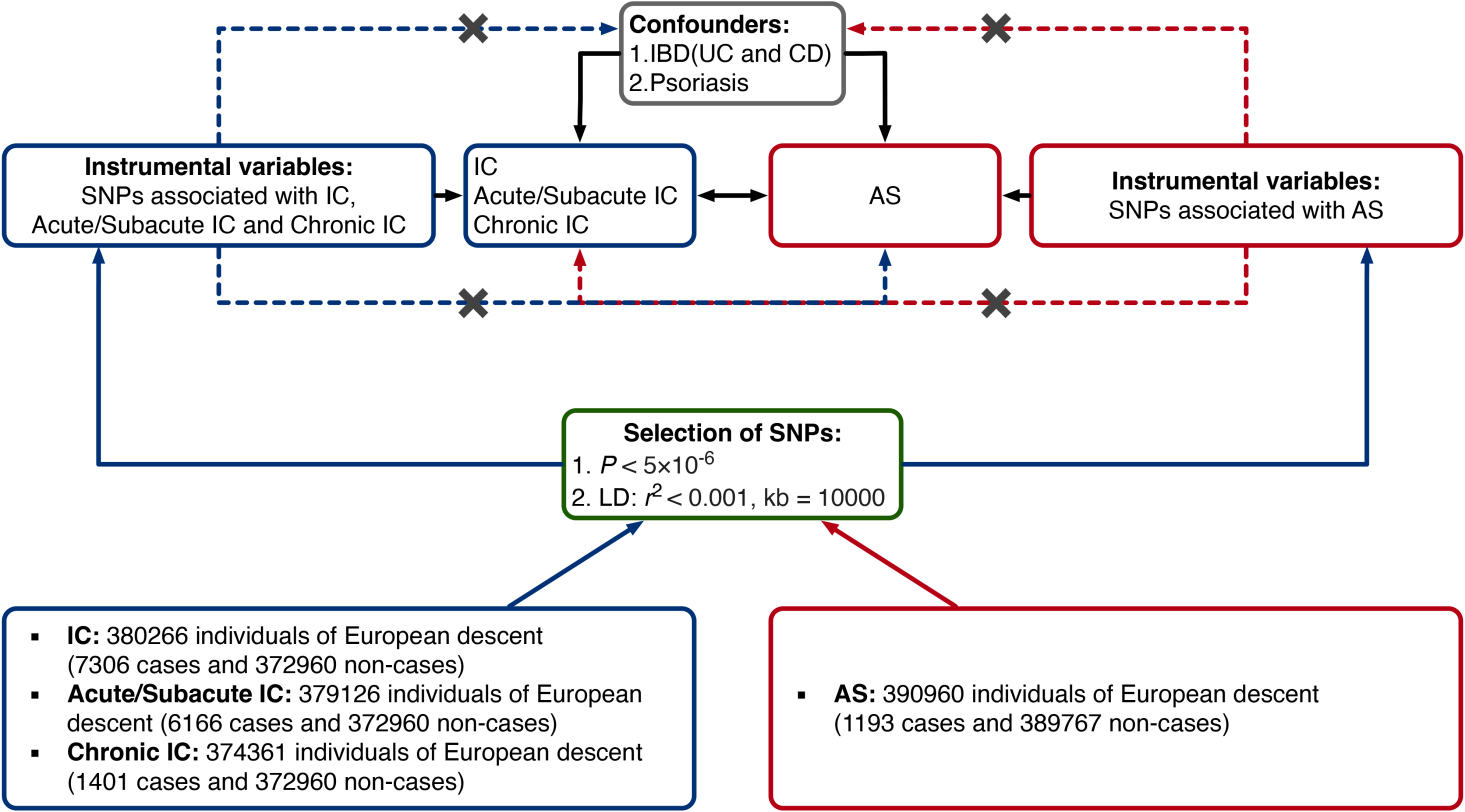
Study design of the present bidirectional MR study of the associations between AS and IC. Black lines show the relationships across the instrumental variables, the exposures, and the outcomes in the MR study examining the bidirectional effects of AS and IC. Dashed lines represent associations that would violate the MR assumptions. AS, ankylosing spondylitis; CD, Crohn’s disease; IBD, inflammatory bowel disease; IC, iridocyclitis; LD, linkage disequilibrium; SNP, single-nucleotide polymorphism; UC, ulcerative colitis.

**Figure 1** provides a concise overview of the bidirectional MR design. MR relies on three fundamental assumptions: (1) IVs are directly associated with the exposure; (2) IVs are not linked to potential confounders; and (3) IVs affect the outcome solely through their influence on the exposure (25).

In our study, we employed two-sample MR to examine bidirectional causal relationships between AS and IC, encompassing IC subtypes - Acute/Subacute IC and Chronic IC.

### 2.2 Data sources

All datasets came from the FinnGen database (http://www.finngen.fi) (v1.15.0 accessed on August 29, 2023) (26), with the study population limited to individuals of European origin to eliminate the bias induced by ethnically related confounding factors. No further ethical approval was necessary because all data were already in the public domain. Summary-level data for IC as a whole (finn-b-H7_IRIDOCYCLITIS) included 7306 cases and 372960 controls. The Acute/Subacute IC data (finn-b-H7_IRIDOACUTE) included 6166 cases and 372960 controls. The Chronic IC data (finn-b-H7_IRIDOCHRONIC) included 1401 cases and 372960 controls. The AS data (finn-b-M13_ANKYLOSPON_STRICT) included 1193 cases and 389767 controls.

### 2.3 Instruments selection

A genome-wide significance level of *P* < 5×10^-6^ and a clumping algorithm with cutoffs of *r* ^2^ = 0.001 (an exception using 0.01 for AS on Acute/Subacute IC to include enough IVs) and kb = 10000 were used to avoid linkage disequilibrium (LD). In the harmonization stage, we excluded palindromic single-nucleotide polymorphisms (SNPs), and if necessary, we conducted a search within the outcome datasets for proxy SNPs with *r* ^2^ value greater than 0.8. Additionally, we implemented the exclusion of outcome-related SNPs with a threshold of *P* < 5×10^-6^. Furthermore, we ensured that no SNPs shared by Acute/Subacute IC and Chronic IC were retained in the reversed MR analysis. Importantly, we retained all SNPs as none had an *F*-statistics less than 10 (**Supplementary Tables 1–6**). To bolster the validity of our instrument selection, we conducted an investigation using PhenoScanner (www.phenoscanner.medschl.cam.ac.uk) on August 30, 2023. PhenoScanner is a comprehensive database of genotype and phenotype relationships in humans (27). Through this analysis, we aimed to identify SNPs connected to potential confounding factors. Notably, our assessment revealed that IBD, including ulcerative colitis and Crohn’s disease (19, 28, 29), and psoriasis (13, 30), had associations with both IC and AS, serving as confounding factors. Consequently, 4 SNPs were excluded (**Supplementary Table 7**).

In our MR analyses of AS as the exposure, we selected 11 SNPs for IC as a whole, 13 SNPs for Acute/Subacute IC, and 8 SNPs for Chronic IC. Conversely, in the reversed MR analyses of IC and its subtypes as the exposures, we selected 9 SNPs of IC as a whole, 15 SNPs of Acute/Subacute IC, and 8 SNPs of Chronic IC for AS. Detailed information about these IVs is provided in **Supplementary Tables 1–6**.

### 2.4 Statistical analysis

The MR pleiotropy residual sum and outlier (MR-PRESSO) test was initially used to detect and remove horizontal pleiotropic outliers. Following this, the remaining IVs underwent further evaluation using the appropriate statistical methods.

To ascertain the causal association, we conducted MR analysis, including the inverse variance weighted (IVW), MR-Egger, and weighted median methods. The IVW method, providing the strongest statistical power, integrates the Wald ratios of the causal effects of each SNP using a meta-analysis approach. MR-Egger regression generates a weighted linear regression of the outcome variables against the exposure variables. The weighted median estimate offers a reliable effect size when at least 50% of the weight in the analysis comes from effective IVs. Causal effect estimates are presented as odds ratios (ORs) with 95% confidence intervals (CIs). Results of the MR analyses were considered significant when the Bonferroni corrected *P*-value was < 0.0083(0.05/6).

To ensure that our MR results adhered to the assumptions, we conducted several sensitivity analyses to investigate the influence of heterogeneity and pleiotropy within the genetic instruments (31). Pleiotropy occurs when a genetic variant affects multiple traits, potentially biasing MR results. We used MR-Egger regression to detect directional pleiotropy, which is indicated by a non-zero intercept. We conducted a heterogeneity test using Cochran’s *Q* test to assess the consistency of IVs with causal effects, which is essential for checking IV assumption violations. If significant heterogeneity (*P*  <  0.05) is observed, the random effects IVW method should be added (32). Finally, the leave-one-out analysis was employed to examine the possible impact of each SNP on an MR estimate by systematically removing one SNP at a time.

All analyses were two-sided and conducted using the TwoSampleMR package (version 0.5.7; https://mrcieu.github.io/TwoSampleMR/) in R software (version 4.2.0; www.r-project.org/).

### 2.5 Power analysis

The power calculation was conducted based on an online tool that considered several parameters, including the sample size of the outcome GWAS, the variance explained by selected SNPs, and the expected effect size, to determine the probability of accurately detecting the causal effects (https://shiny.cnsgenomics.com/mRnd/) (33).

## 3 Results

### 3.1 Associations of genetic liability to IC with the risk of AS

**Figure 2:**
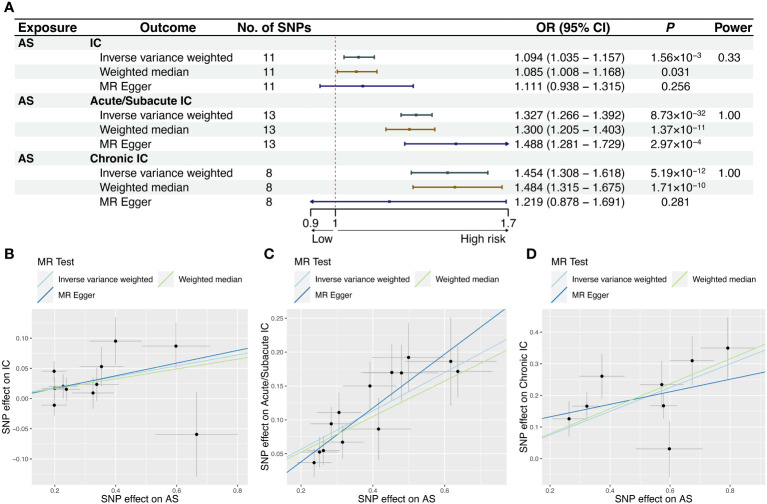
MR results and scatter plots of causal estimates for AS on IC and its subtypes. Scatter plot: The slope of each line corresponds to the causal estimates for each method. Individual SNP effect on the outcome (point and vertical line) against its effect on the exposure (point and horizontal line) is delineated in the background. **(A)** MR results of causal estimates for AS on IC and its subtypes. Scatter plots of causal estimates for AS on **(B)** IC; **(C)** Acute/Subacute IC; **(D)** Chronic IC. AS, ankylosing spondylitis; CI, confidence interval; IC, iridocyclitis; MR, Mendelian randomization; OR, odds ratio; SNP, single-nucleotide polymorphism.

Our study revealed a causal relationship between AS and an increased risk of IC in the European population. The MR-PRESSO analysis did not identify any distorted outliers. We utilized the IVW method to illustrate the associations between AS and the risk of IC as a whole (OR = 1.094, 95% CI = 1.035-1.157, *P* = 0.00156), Acute/Subacute IC (OR = 1.327, 95% CI = 1.266-1.392, *P* = 8.73×10^-32^) and Chronic IC (OR = 1.454, 95% CI = 1.308-1.618, *P* = 5.19×10^-12^). The forest plots displaying the causal relationships between genetically predicted AS with the risk of IC and its subtypes can be seen in **Figure 2A**. The scatter plots depicting the causal relationships from the MR analysis are presented in **Figures 2B–D**. These associations remained consistent across various sensitivity analyses, despite the lack of significance in the MR-Egger regression analysis (**Figure 2A**). The forest plots, which demonstrate the causal effects of each SNP, are provided in **Supplementary Figure 1**. Heterogeneity was not detected by Cochran’s *Q* value, and MR-Egger regression did not yield evidence of non-balanced pleiotropy among individual SNP estimates (**Table 1**, **Supplementary Figure 2**). The leave-one-out analysis confirmed that no single SNP significantly drove the observed association (**Supplementary Figure 3**).

**Table 1:** Heterogeneity and Pleiotropy tests.

Exposure	Outcome	No. of SNPs	Heterogeneity test		MR-Egger pleiotropy test	
			*Q*	*P*	Intercept	*P*
**AS**	IC	11	12.451	0.256	-0.004	0.861
**AS**	Acute/Subacute IC	13	9.742	0.639	-0.041	0.144
**AS**	Chronic IC	8	11.265	0.127	0.093	0.308
**IC**	AS	9	9.410	0.309	-0.037	0.504
**Acute/Subacute IC**	AS	15	36.852	7.77×10^-4^	0.009	0.726
**Chronic IC**	AS	8	5.047	0.654	-0.009	0.859

AS, ankylosing spondylitis; IC, iridocyclitis; MR, Mendelian randomization; SNP, single-nucleotide polymorphism.

### 3.2 Associations of genetic liability to AS with the risk of IC

**Figure 3:**
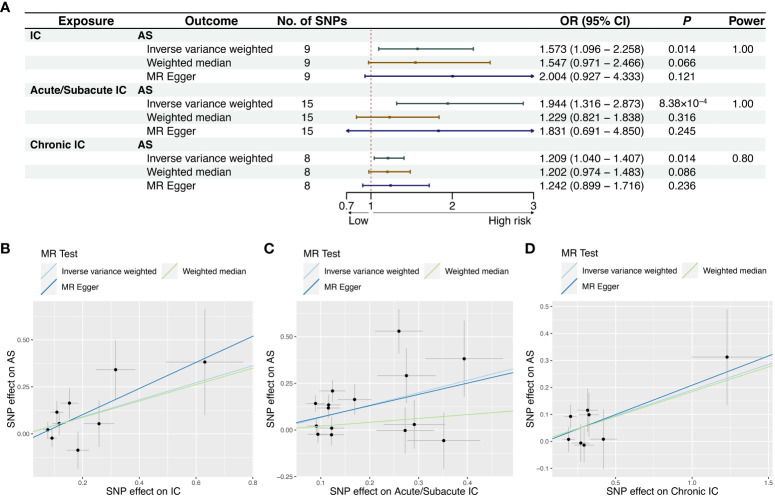
MR results and scatter plots of causal estimates for IC and its subtypes on AS. **(A)** MR results of causal estimates for IC and its subtypes on AS. Scatter plots of causal estimates for **(B)** IC; **(C)** Acute/Subacute IC; **(D)** Chronic IC on AS. AS, ankylosing spondylitis; CI, confidence interval; IC, iridocyclitis; MR, Mendelian randomization; OR, odds ratio; SNP, single-nucleotide polymorphism.

The reverse MR study aimed to explore the causal relationship between IC and its subtypes with AS. After excluding one outlier (rs78933533) identified through MR-PRESSO, the IVW analysis indicated that IC as a whole was not significantly associated with the risk of AS (OR = 1.573, 95% CI = 1.096-2.258, *P* = 0.0141). However, further analysis revealed a causal relationship between Acute/Subacute IC and AS (OR = 1.944, 95% CI = 1.316-2.873, *P* = 8.38×10^-4^), whereas Chronic IC did not exhibit a significant causal effect (OR = 1.209, 95% CI = 1.040-1.407, *P* = 0.0136). The forest plots illustrating the causal relationships between genetically predicted IC and its subtypes with the risk of AS are shown in **Figure 3A**. The scatter plots exhibiting the causal relationships from the MR analysis are shown in **Figures 3B–D**. Sensitivity analyses provided support for these associations, even though they did not reach statistical significance in the MR-Egger regression analysis (**Figure 3A**). **Supplementary Figure 1** offers forest plots detailing the causal effect of each SNP. The MR-Egger regression did not reveal directional pleiotropy in the impact of IC and its subtypes on AS. Cochran’s *Q* test revealed significant heterogeneity among SNPs in Acute/Subacute IC (*Q*=36.852, *P* = 7.77×10^-4^) (**Table 1**, **Supplementary Figure 2**). We conducted an additional MR analysis using the random effects IVW method, which confirmed consistent causal effect estimates (**Supplementary Table 8**). The leave-one-out analysis verified that no single SNP had a substantial impact on the observed association (**Supplementary Figure 3**).

### 3.3 Power calculation

Power calculation was conducted online, suggesting that the IVs provided accurate estimates of causal effects (**Figures 2A**, **3A**).

## 4 Discussion

Our findings underscore the intricate relationship between AS and IC, with varying associations observed across different subtypes of IC. The results support a robust causal relationship between AS and IC, particularly in the case of Acute/Subacute IC on AS, within the European population.

The potential correlation between AS and IC has been suggested by previous observational studies. However, establishing causality from such studies is challenging due to potential confounding variables and the risk of reverse causality. Randomized controlled trials (RCTs) are considered the gold standard for establishing causality, but they are often costly, time-consuming, and may not always be feasible for testing specific hypotheses (25). Notably, there hasn’t been a randomized, controlled study specifically designed to investigate the relationship between AS and IC. Therefore, it remains unclear whether an association exists between AS and IC and, if so, the direction of this association. Bidirectional MR provides an efficient alternative to address these limitations.

Our initial MR analysis aimed to uncover the association between AS and the risk of IC. Previous observational studies have suggested the possibility of IC occurring as a secondary condition to AS. A systematic review and meta-analysis of 143 studies involving 44372 AS patients found that uveitis had a pooled prevalence of 25.8% (95% CI = 24.1%-27.6%) and was positively correlated with disease duration (β = 0.05, 95% CI = 0.03-0.08) (13). A pooled random effects model found uveitis in 24% of adult AS patients across 23 studies with 11943 individuals (34). Additionally, a meta-analysis of 8 observational studies revealed a higher pooled prevalence of uveitis in AS (23.0%) compared to non-radiographic axial spondyloarthritis (15.9%) (35). Our findings align with previous investigations and confirm that AS is indeed associated with an increased risk of IC as a whole (OR = 1.094, *P* = 0.00156), Acute/Subacute IC (OR = 1.327, *P* = 8.73×10^-32^), and Chronic IC (OR = 1.454, *P* = 5.19×10^-12^). These results further strengthen the evidence for a causal relationship between AS and a higher risk of IC.

In our reverse MR analysis, we sought to investigate the potential association between IC and the risk of AS. Previous observational studies have indicated that IC might play a significant role in leading to a diagnosis of AS. A population-based study involving 4101 AS patients found that 11.4% of them had IC at the time of their AS diagnosis, indicating a significant association (HR = 15, 95% CI = 11.6-20.7) (14). Another nationwide cohort study with 10483 patients demonstrated an increased risk of AS in those with anterior uveitis (RR = 7.40, 95% CI= 4.99-10.98) (36). Furthermore, a retrospective cohort analysis of 2097 AS patients reported a 45.1% occurrence of uveitis across various cohorts (37). Data from the TReasure database confirmed that 11.0% of AS patients had experienced at least one episode of uveitis (38). A comparison study utilizing high-density genotyping revealed a substantial difference in the effect magnitude between AS patients with and without IC (39). Despite these observations, our comprehensive MR analysis found no significant association between genetic susceptibility to IC as a whole and an elevated risk of AS. However, when we examined IC subtypes, we discovered a significant association between Acute/Subacute IC and AS (OR = 1.944, *P* = 8.38×10^-4^). In contrast, Chronic IC did not exert a causal effect on AS, aligning with previous findings that suggested a lower likelihood of IC remission with diagnosis of spondyloarthropathy (40). These results provide nuanced insights into the relationship between IC and AS, highlighting the importance of considering IC subtypes when exploring their potential causal links.

This study expands on previous research by establishing a causal relationship between Acute/Subacute IC, as opposed to IC as a whole or Chronic IC, and AS. While the exact pathophysiological mechanisms underpinning this association remain incompletely understood, there is growing consensus that AS and IC likely share a common etiology. Proposed explanations for this link include shared genetic susceptibility loci (39, 41), immune system dysfunction (15, 42), disruptions in gut microbiota (43, 44), and environmental factors (14, 45). Notably, our findings differ slightly from earlier estimates, possibly due to variations in analytical approaches. Inevitable clinical confounding factors may influence observational research, making it more challenging to draw definitive conclusions about causality. These confounding factors can affect both the exposure and the outcome. Therefore, even if observational studies identify a significant correlation, they cannot establish direct causation. Mendelian randomization helps circumvent these issues by integrating genetic instrumental variables. To ensure the robustness and consistency of our causal estimates, we conducted sensitivity analyses, further strengthening the reliability of our findings.

We acknowledge several limitations in our study. Firstly, sample overlap may occur as both the AS and IC GWASs originated from the FinnGen study. However, recent studies have demonstrated the viability of using the two-sample MR method for large datasets with sample sizes exceeding 300000 from a single source (46). Additionally, we computed the *F* statistic as a metric to assess the robustness and efficacy of the IVs in our analyses, and all IVs’ *F*-values exceeded 10, providing further confidence in the reliability of our analysis (47). To assess the potential overlap bias, we used an online tool based on a relevant study (https://sb452.shinyapps.io/overlap/) to calculate the overlap bias (48). The results showed that the bias values in all groups were 0.000, even under a 100% overlap assumption, with a type I error rate of 0.05 (**Supplementary Figure 4**, **Supplementary Table 9**). This suggests that demographic overlap is less likely to skew our results. Secondly, the lower *P* threshold was established due to the paucity of IVs. Thirdly, although our *F*-statistic tests did not indicate significant instrument bias, cautious interpretation is still advisable. Fourthly, our findings, focused on individuals of European heritage, may not be directly applicable to other racial or ethnic groups. Lastly, our conclusion is based on genetic predisposition and does not consider other factors such as environmental influences and treatment status that may contribute to the development of IC or AS. For example, once AS is diagnosed, patients often undergo immunomodulatory therapy, which can reduce the probability of IC occurrence to some extent. On the other hand, individuals with IC, especially those with Acute/Subacute IC, are often screened for HLA-B27 at the onset of the disease, which increases the detection rate of IC-related AS. As more comprehensive information including clinical profiles, test results, and treatment status for the individuals in the FinnGen database becomes available in the future, further research is needed to obtain a more thorough understanding of the relationships between AS and IC.

## Conclusion

In this MR study, we established a causal relationship between IC, especially the Acute/Subacute subtype, and an increased risk of AS. Notably, Chronic IC did not demonstrate a causal effect on AS. Conversely, AS is causally associated with the development of IC and its subtypes. These findings have clinical implications for identifying AS risk in IC patients and tailoring management. Further research is warranted to explore the underlying mechanisms.

## Data availability statement

The datasets used and analyzed in this study can be found at http://www.finngen.fi/. Further information is available from the corresponding author upon reasonable request.

## Author contributions

HL: Conceptualization, Data curation, Formal analysis, Methodology, Software, Writing – original draft. YX: Conceptualization, Methodology, Writing – original draft. QG: Conceptualization, Methodology, Writing – original draft. TZ: Conceptualization, Methodology, Writing – original draft. SZ: Data curation, Formal analysis, Software, Writing – original draft. MW: Data curation, Formal analysis, Software, Writing – original draft. YC: Funding acquisition, Supervision, Writing – review & editing. CG: Funding acquisition, Supervision, Writing – review & editing.
