# Supplementary material for: Ankylosing spondylitis: acute/subacute vs. chronic iridocyclitis - a bidirectional two-sample Mendelian randomization study
Source: Front Immunol. 2024 Jan 11;14:1295118. doi: 10.3389/fimmu.2023.1295118 (PMC10808375; doi:10.3389/fimmu.2023.1295118)
Supplement: Supplementary file 1 [file DataSheet_1.docx]

Supplementary Material

# Supplementary Figures

**
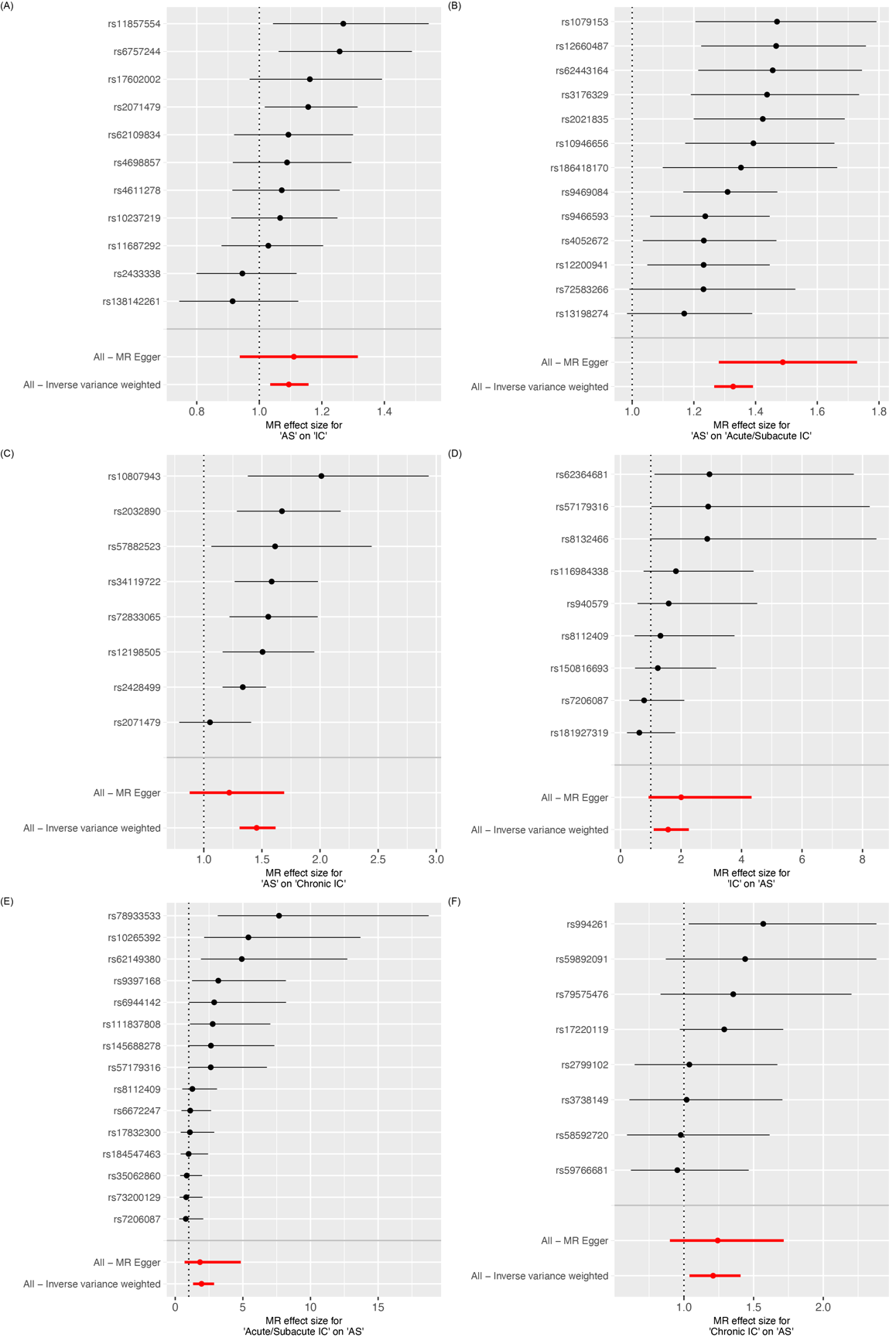
**

**Supplementary Figure 1. Forest plots of causal relationships between AS and IC.**

Forest plot: The dot and bar indicate the causal estimate of exposure on the risk of outcome. Forest plot of causal estimates for **(A)** AS on IC; **(B)** AS on Acute/Subacute IC; **(C)** AS on Chronic IC; **(D)** IC on AS; **(E)** Acute/Subacute IC on AS; **(F)** Chronic IC on AS. AS, ankylosing spondylitis; IC, iridocyclitis; MR, Mendelian randomization.

**

**

**Supplementary Figure 2. Funnel plots in heterogeneity analysis of causal estimates for each genetic variant.**

Funnel plot: The light blue line represents the inverse-variance weighted estimate, and the dark blue line represents the MR‐Egger estimate. Funnel plot in heterogeneity analysis of genetic associations with **(A)** AS against those with IC; **(B)** AS against those with Acute/Subacute IC; **(C)** AS against those with Chronic IC; **(D)** IC against those with AS; **(E)** Acute/Subacute IC against those with AS; **(F)** Chronic IC against those with AS. AS, ankylosing spondylitis; β, beta coefficient; IC, iridocyclitis; IV, instrumental variable; MR, Mendelian randomization; SE, standard error.





**Supplementary Figure 3. The leave-one-out analysis of a single SNP in the exposures for the outcomes.**

The leave-one-out analysis: Each black point in the forest plot represents the MR analysis by the inverse variance weighted method excluding that particular SNP. **The l**eave-one-out analysis of single SNP of **(A)** AS for IC; **(B)** AS for Acute/Subacute IC; **(C)** AS for Chronic IC; **(D)** IC for AS; **(E)** Acute/Subacute IC for AS; **(F)** Chronic IC for AS. AS, ankylosing spondylitis; IC, iridocyclitis; MR, Mendelian randomization; SNP, single-nucleotide polymorphism.

**Supplementary Figure 4. Bias and Type 1 error rate of Mendelian randomization with sample overlap.**

# Supplementary Tables

**Supplementary Table 1. MR results of a single SNP in AS for IC.**

| SNPs | Effect allele | Other allele | Beta | SE | EAF | *P* | *F* |
| --- | --- | --- | --- | --- | --- | --- | --- |
| rs10237219 | T | G | 0.238 | 0.045 | 0.310 | 1.30E-07 | 9694 |
| rs11687292 | T | C | -0.326 | 0.069 | 0.087 | 2.15E-06 | 6692 |
| rs11857554 | T | G | 0.400 | 0.086 | 0.067 | 3.47E-06 | 8010 |
| rs138142261 | A | G | 0.666 | 0.136 | 0.029 | 9.53E-07 | 9891 |
| rs17602002 | C | T | 0.353 | 0.072 | 0.099 | 9.21E-07 | 8926 |
| rs2071479 | T | C | -0.598 | 0.111 | 0.027 | 7.79E-08 | 7579 |
| rs2433338 | C | T | 0.198 | 0.042 | 0.604 | 1.84E-06 | 7487 |
| rs4611278 | A | G | -0.338 | 0.073 | 0.076 | 3.35E-06 | 6326 |
| rs4698857 | T | G | 0.199 | 0.042 | 0.392 | 2.04E-06 | 7548 |
| rs62109834 | A | G | 0.227 | 0.047 | 0.271 | 1.18E-06 | 8153 |
| rs6757244 | C | A | -0.198 | 0.041 | 0.537 | 1.48E-06 | 7750 |

**Supplementary Table 2. MR results of a single SNP in AS for Acute/Subacute IC.**

| SNPs | Effect allele | Other allele | Beta | SE | EAF | *P* | *F* |
| --- | --- | --- | --- | --- | --- | --- | --- |
| rs1079153 | C | T | 0.499 | 0.102 | 0.049 | 1.02E-06 | 8887 |
| rs10946656 | A | G | -0.284 | 0.058 | 0.127 | 1.17E-06 | 6855 |
| rs12200941 | G | T | 0.262 | 0.046 | 0.290 | 9.49E-09 | 10771 |
| rs12660487 | G | T | 0.392 | 0.075 | 0.090 | 1.78E-07 | 9574 |
| rs13198274 | C | A | -0.236 | 0.048 | 0.214 | 8.92E-07 | 7224 |
| rs186418170 | T | A | 0.617 | 0.127 | 0.034 | 1.29E-06 | 9519 |
| rs2021835 | T | C | -0.480 | 0.102 | 0.034 | 2.72E-06 | 5812 |
| rs3176329 | G | T | -0.306 | 0.062 | 0.865 | 8.74E-07 | 8327 |
| rs4052672 | A | C | -0.252 | 0.052 | 0.170 | 1.61E-06 | 6867 |
| rs62443164 | T | C | 0.453 | 0.086 | 0.069 | 1.28E-07 | 10059 |
| rs72583266 | C | T | 0.416 | 0.091 | 0.061 | 4.67E-06 | 7547 |
| rs9466593 | A | G | -0.316 | 0.059 | 0.121 | 9.44E-08 | 8131 |
| rs9469084 | T | C | -0.637 | 0.096 | 0.035 | 3.87E-11 | 10465 |

**Supplementary Table 3. MR results of a single SNP in AS for Chronic IC.**

| SNPs | Effect allele | Other allele | Beta | SE | EAF | *P* | *F* |
| --- | --- | --- | --- | --- | --- | --- | --- |
| rs10807943 | C | T | -0.373 | 0.075 | 0.911 | 7.79E-07 | 8625 |
| rs12198505 | C | T | 0.572 | 0.073 | 0.101 | 3.91E-15 | 21789 |
| rs2032890 | C | A | -0.323 | 0.048 | 0.207 | 1.91E-11 | 12912 |
| rs2071479 | T | C | -0.598 | 0.111 | 0.027 | 7.79E-08 | 7293 |
| rs2428499 | A | G | -0.578 | 0.045 | 0.197 | 3.55E-37 | 36910 |
| rs34119722 | C | G | 0.674 | 0.074 | 0.100 | 7.61E-20 | 29399 |
| rs57882523 | T | C | 0.263 | 0.056 | 0.169 | 2.95E-06 | 7461 |
| rs72833065 | C | T | 0.792 | 0.091 | 0.066 | 3.27E-18 | 28020 |

**Supplementary Table 4: MR results of a single SNP in IC for AS.**

| SNPs | Effect allele | Other allele | Beta | SE | EAF | *P* | *F* |
| --- | --- | --- | --- | --- | --- | --- | --- |
| rs116984338 | T | C | -0.631 | 0.136 | 0.003 | 3.64E-06 | 928 |
| rs150816693 | T | C | -0.258 | 0.054 | 0.023 | 1.54E-06 | 1151 |
| rs181927319 | C | T | 0.183 | 0.039 | 0.053 | 2.13E-06 | 1275 |
| rs57179316 | T | C | 0.153 | 0.032 | 0.078 | 1.52E-06 | 1291 |
| rs62364681 | T | G | -0.316 | 0.069 | 0.014 | 4.29E-06 | 1030 |
| rs7206087 | C | T | -0.092 | 0.019 | 0.715 | 9.00E-07 | 1309 |
| rs8112409 | A | G | -0.077 | 0.017 | 0.461 | 4.21E-06 | 1128 |
| rs8132466 | T | C | 0.109 | 0.024 | 0.152 | 4.55E-06 | 1167 |
| rs940579 | G | T | -0.117 | 0.025 | 0.862 | 2.01E-06 | 1239 |

**Supplementary Table 5. MR results of a single SNP in Acute/Subacute IC for AS.**

| SNPs | Effect allele | Other allele | Beta | SE | EAF | *P* | *F* |  |
| --- | --- | --- | --- | --- | --- | --- | --- | --- |
| rs10265392 | T | C | -0.124 | 0.027 | 0.859 | 3.59E-06 | 1403 | |
| rs111837808 | A | G | 0.116 | 0.024 | 0.193 | 1.11E-06 | 1572 | |
| rs145688278 | A | G | 0.393 | 0.080 | 0.015 | 7.97E-07 | 1773 | |
| rs17832300 | T | G | 0.122 | 0.026 | 0.146 | 3.03E-06 | 1410 | |
| rs184547463 | G | C | -0.273 | 0.059 | 0.022 | 3.89E-06 | 1234 | |
| rs35062860 | T | C | -0.351 | 0.075 | 0.013 | 2.68E-06 | 1236 | |
| rs57179316 | T | C | 0.169 | 0.035 | 0.079 | 9.87E-07 | 1580 | |
| rs62149380 | T | G | -0.089 | 0.019 | 0.335 | 3.27E-06 | 1341 | |
| rs6672247 | G | A | -0.291 | 0.063 | 0.020 | 3.34E-06 | 1235 | |
| rs6944142 | A | G | -0.275 | 0.060 | 0.974 | 4.01E-06 | 1457 | |
| rs7206087 | C | T | -0.094 | 0.020 | 0.715 | 3.82E-06 | 1365 | |
| rs73200129 | T | C | -0.122 | 0.026 | 0.143 | 1.99E-06 | 1379 | |
| rs78933533 | A | G | -0.260 | 0.049 | 0.033 | 9.59E-08 | 1629 | |
| rs8112409 | A | G | -0.091 | 0.018 | 0.458 | 6.36E-07 | 1552 | |
| rs9397168 | G | A | 0.116 | 0.024 | 0.178 | 1.47E-06 | 1489 | |

**Supplementary Table 6. MR results of a single SNP in Chronic IC for AS.**

| SNPs | Effect allele | Other allele | Beta | SE | EAF | *P* | *F* |
| --- | --- | --- | --- | --- | --- | --- | --- |
| rs17220119 | G | A | -1.229 | 0.233 | 0.004 | 1.25E-07 | 4417 |
| rs2799102 | C | A | 0.192 | 0.041 | 0.311 | 3.17E-06 | 5803 |
| rs3738149 | T | C | 0.420 | 0.089 | 0.053 | 2.34E-06 | 6524 |
| rs58592720 | A | G | 0.272 | 0.059 | 0.123 | 4.38E-06 | 5862 |
| rs59766681 | A | G | -0.293 | 0.063 | 0.088 | 3.43E-06 | 5107 |
| rs59892091 | C | T | 0.316 | 0.068 | 0.092 | 3.17E-06 | 6156 |
| rs79575476 | C | G | 0.325 | 0.067 | 0.094 | 1.35E-06 | 6637 |
| rs994261 | C | T | 0.205 | 0.040 | 0.697 | 4.25E-07 | 6497 |

# Supplementary Table 7. Deleted SNPs associated with confounders in PhenoScanner

| SNPs | Effect allele | Other allele | Traits | *P* |
| --- | --- | --- | --- | --- |
| rs2011743 | C | T | Inflammatory bowel disease | 2.31E-06 |
| rs2011743 | C | T | Ulcerative colitis | 4.99E-06 |
| rs296563 | G | T | Inflammatory bowel disease | 3.43E-11 |
| rs296563 | G | T | Ulcerative colitis | 3.29E-08 |
| rs2847293 | A | T | Crohns disease | 7.98E-13 |
| rs2847293 | A | T | Inflammatory bowel disease | 7.22E-11 |
| rs34236350 | C | T | Inflammatory bowel disease | 1.56E-10 |
| rs34236350 | C | T | Ulcerative colitis | 1.38E-11 |
| rs3767501 | C | T | Inflammatory bowel disease | 3.83E-09 |
| rs3767501 | C | T | Ulcerative colitis | 1.30E-08 |

**Supplementary Table 8. MR result using random effects IVW method for Acute/Subacute IC and AS.**

| **Exposure** | **Outcome** | **NSNP** | **Beta** | **SE** | ***P*** | **OR (95%CI)** |
| --- | --- | --- | --- | --- | --- | --- |
| Acute/Subacute IC | AS | 15 | 0.665 | 0.199 | 0.000838 | 1.944(1.316-2.873) |

**Supplementary Table 9. Detailed information for Bias and Type 1 error rate of MR with sample** **overlap.**

| **Exposure** | **Outcome** | **Overlap(%)** | **Bias** | **Type 1 error rate** |
| --- | --- | --- | --- | --- |
| AS | IC | 5.73 | 0.000 | 0.05 |
| AS | Acute/Subacute IC | 6.09 | 0.000 | 0.05 |
| AS | Chronic IC | 4.41 | 0.000 | 0.05 |
| IC | AS | 5.73 | 0.000 | 0.05 |
| Acute/Subacute IC | AS | 6.09 | 0.000 | 0.05 |
| Chronic IC | AS | 4.41 | 0.000 | 0.05 |

AS, ankylosing spondylitis; Beta, beta coefficient; CI, confidence interval; EAF, effect attributable to the factor; *F*, *F* statistic; IC, iridocyclitis; IVW, inverse variance weighted; OR, odds ratio; SE, standard error; SNP, single-nucleotide polymorphism.
